# Effects of coal-fired PM_2.5_ on the expression levels of atherosclerosis-related proteins and the phosphorylation level of MAPK in ApoE^−/−^ mice

**DOI:** 10.1186/s40360-020-00411-8

**Published:** 2020-05-08

**Authors:** Siqi Wang, Feifei Wang, Lixin Yang, Qin Li, Yao Huang, Zhiyuan Cheng, Hongqian Chu, Yiming Song, Lanqin Shang, Weidong Hao, Xuetao Wei

**Affiliations:** 1 Department of Toxicology, School of Public Health, Peking University Health Science Center, No.38 XueYuan Road, HaiDian District, Beijing, 100191 People’s Republic of China; 2 Beijing Key Laboratory of Toxicological Research and Risk Assessment for Food Safety, Beijing, 100191 People’s Republic of China; 3 State Key Laboratory of Environmental Criteria and Risk Assessment, Chinese Research Academy of Environmental Sciences, Beijing, 100012 People’s Republic of China; 4 Translational Medicine Center, Beijing Chest Hospital, Capital Medical University, Beijing, 101149 People’s Republic of China; 5 Beijing Key Laboratory in Drug Resistant Tuberculosis Research, Beijing Tuberculosis and Thoracic Tumor Research Institute, Beijing, 101149 People’s Republic of China

**Keywords:** ApoE^−/−^ mice, Atherosclerosis, Coal-fired PM_2.5_

## Abstract

**Background:**

Air pollution increases the morbidity and mortality of cardiovascular disease (CVD). Atherosclerosis (AS) is the pathological basis of most CVD, and the progression of atherosclerosis and the increase of fragile plaque rupture are the mechanism basis of the relationship between atmospheric particulate pollution and CVD. The aim of the present study was to investigate the effects of coal-fired fine particulate matter (PM_2.5_) on the expression levels of atherosclerosis-related proteins (von Willebrand factor (vWF), Endothelin-1 (ET-1), intercellular adhesion molecule-1 (ICAM-1), and E-selectin, and to explore the role and mechanism of the progression of atherosclerosis induced by coal-fired PM_2.5_ via the mitogen-activated protein kinase (MAPK) signaling pathways.

**Methods:**

Different concentrations of PM_2.5_ were given to apolipoprotein-E knockout (ApoE^−/−^) mice via intratracheal instillation for 8 weeks. Enzyme-linked immunosorbent assay (ELISA) was used to detect the levels of vWF, ET-1 in serum of mice. Immunohistochemistry was used to observe the expression and distribution of ICAM-1 and E-selectin in the aorta of mice. Western blot was used to investigate the phosphoylation of proteins relevant to MAPK signaling pathways.

**Results:**

Coal-fired PM_2.5_ exacerbated atherosclerosis induced by a high-fat diet. Fibrous cap formation, foam cells accumulation, and atherosclerotic lesions were observed in the aortas of PM_2.5_-treated mice. Coal-fired PM_2.5_ increased the protein levels of ET-1, ICAM-1, and E-selectin, but there was no significant difference in the vWF levels between the PM_2.5_-treatment mice and the HFD control mice. Coal-fired PM_2.5_ promoted the phosphorylation of p38, c-Jun N-terminal kinase (JNK), extracellular signal-regulated kinase (ERK) in aortic tissues of mice.

**Conclusion:**

Coal-derived PM_2.5_ exacerbated the formation of atherosclerosis in mice, increased the expression levels of atherosclerosis-related proteins in mice serum, and promoted the phosphorylation of proteins relevant to MAPK signaling pathway. Thus, MAPK signaling pathway may play a role in the atherosclerosis pathogenesis induced by Coal-derived PM_2.5_.

## Background

Air pollution caused 4.1 million deaths globally in 2016 alone and is the sixth highest-ranking risk factor for global mortality [1]. Air pollution increases the morbidity and mortality of cardiovascular disease (CVD) [2–5]. According to a report of the World Health Organization (WHO), around 17.3 million people die of CVD each year, which accounts for 30% of all deaths [6]. Fine particulate matter (PM_2.5_) is the main toxic component of atmospheric particulate matter and primarily derives from coal-fired emission and automobile exhaust [7]. Moreover, an estimated 2.9 million deaths was attributed to PM_2.5_ in 2013 alone, which is considered as a leading risk factor for global disease [8]. PM_2.5_ is one of the primary causes of death in China and accounted for 11.1% of all deaths in China in 2016 [1]. Since the PM_2.5_ pollution is mainly caused by coal-burning emission in China [9, 10], it is important for future research to further elucidate the effects and mechanisms of coal-fired PM_2.5_ on cardiovascular diseases.

Atherosclerosis (AS) is a type of CVD [11] and is one of the leading causes of death around the world [12–14]. AS is also the pathological basis of most CVD, and the progression of atherosclerosis and the increase of fragile plaque rupture are the mechanism basis of the relationship between atmospheric particulate pollution and CVD [15–17]. PM_2.5_ increased mortality in individuals with CVD [18] via its contribution to the development of atherosclerosis [19]. Additionally, exposure to PM_2.5_ is a fundamental cause of cardiovascular diseases [20].

PM_2.5_ has short-term and long-term effect on cardiovascular system [14, 21]. Animal studies have suggested that PM_2.5_ exposure increases both the area of atherosclerotic plaques and plaque vulnerability in apolipoprotein-E knockout (ApoE^−/−^) mice and rats [19, 22, 23]. Similar results have been found from epidemiological studies, which have revealed that exposure to PM_2.5_ increases the development of atherosclerosis in humans [24–26]. PM_2.5_ presumably exerts atherogenic effects by inducing endothelial damage, mitochondrial injury, inflammatory responses, and oxidative stress [23, 27, 28]. However, the underlying molecular processes and potential mechanisms remain to be fully elucidated due to the etiological complexity of the atherogenesis [29, 30].

Mitogen-activated protein kinase (MAPK) signaling pathways are a series of parallel cascades of serine/threonine kinase, including extracellular signal-regulated kinase (ERK), c-Jun N-terminal kinase (JNK), and p38 MAPK [31]. MAPK signaling pathways play a key role in the atherosclerosis development [32], and it deserves further investigation in PM_2.5_-induced atherosclerosis. Thus, the present study aimed at exploring the mechanisms of atherosclerosis induced by coal-fired PM_2.5_ and the role of MAPK signaling pathways in this disease progression.

## Methods

### Coal-fired PM_2.5_ collection and extraction

Raw coal from a typical coal field (Yinchuan) in China was purchased from state-owned coal mines. The coal samples were broken into pieces and ignited in the stove. PM_2.5_ emitted from coal combustion was sampled by the dilution tunnel system, and dilution and sampling continued until the combustion finished [33].

The PM_2.5_ filters were extracted with ultra-pure water in an ultrasonic bath. After ultrasonic elution and freeze-drying, coal-fired PM_2.5_ suspensions were prepared and stored at − 20 °C until they were used for exposure to mice.

### Animals and experimental groups

ApoE^−/−^ C57BL/6 J mice represent a common experimental model for atherosclerosis research. Forty ApoE^−/−^ male mice (7–8 weeks old, weight ranged from 18 g to 20 g) were obtained from Beijing Vital River Laboratory Animal Technology Co., Ltd. Mice were housed in a barrier system at a controlled temperature (22 ± 2 °C) and a relative humidity 40–70%, with a 12 h:12 h light:dark cycle. All animals were given free access to food and water. Atherosclerosis model groups were fed with a high-fat diet (HFD) consisting of 54% regular chow, 20% sugar, 15% lard oil, 7.8% casein, 1.7% calcium hydrogen phosphate, 1.2% cholesterol, and 0.2% bile salt.

Choosing 6 to 10 mice for each group in general mice experiments to meet the statistical requirements, and we chose the median 8. It not only avoided sample size reduction caused by accidental death during the experiment, but also followed the rules of 3R which contain the reduction of animal usage. After 1 week of acclimatization, mice were divided into the following five groups randomly by using random number table (*n* = 8) and treated with PM_2.5_ or phosphate buffer saline (PBS): (1) normal control group (normal diet + PBS); (2) HFD control group (HFD + PBS); (3) low-dose group (HFD + PM_2.5_ 0.05 mg/kg of body weight [bw]/week); (4) middle-dose group (HFD + PM_2.5_ 0.50 mg/kg of bw/week); and (5) high-dose group (HFD + PM_2.5_ 5.00 mg/kg of bw/week). Basing on previously reported study [34] and our laboratory former work, different concentrations of coal-fired PM_2.5_ (0, 0.05, 0.50, and 5.00 mg/kg of bw) were given to ApoE^−/−^ mice once a week (at 8.00–11.00 a.m. of Tuesday) via intratracheal instillation. After 8 weeks treatment [21], mice were sacrificed by cervical dislocation under isoflurane anaesthesia. Whole-blood samples kept at room temperature for 30 min after they were collected through aortas, and then they were centrifuged at 3000 g for 10 min. Sera were collected and stored at − 80 °C. Aortic root samples were fixed in 4% paraformaldehyde and embedded in paraffin, after which they were used for histopathological and immunohistochemical analyses.

### Histopathology

As previously reported [35, 36], aortas isolated from all groups were fixed in 4% paraformaldehyde for 48 h and subsequently embedded in paraffin. For histopathological assessment, all samples of aorta root were processed into serial sections with 6-μm thick, and stained with hematoxylin and eosin (H&E).

### Immunohistochemistry

Immunohistochemistry was performed as previously described [37]. The paraffin-embedded tissue sections were dewaxed, then immersed in 0.01 mol/L citric acid buffer and heated to boiling in an autoclave for 2 min. After treated with 0.3% hydrogen peroxide solution for 10 min, the tissue sections were blocked with 5% bovine serum albumin (BSA) for 1 h at 37 °C. Then they were washed three times with tris buffered saline (TBS), and were then incubated with primary antibodies (rat anti-mouse intercellular adhesion molecule-1 [ICAM-1] [dilution 1:100] and rabbit anti-mouse E-selectin [dilution 1:25], both purchased from Abcam, UK) for 2 h at 37 °C. The tissue sections were then washed three times with TBS, and treated with appropriate horseradish peroxidase (HRP)- conjugated secondary antibodies for 2 h at 37 °C. Then they were then rinsed three times with TBS again. Antigen-antibody reactions were stained with diaminobenzidine (DAB), and sections were also counterstained with H&E. The expression levels of ICAM-1 and E-selectin were observed with a Nikon E400 microscope under high-power (400×) fields.

### Enzyme-linked immunosorbent assays (ELISAs)

The concentrations of Endothelin-1 (ET-1) and von Willebrand factor (vWF) in the mice sera were determined by ELISA kits according to the manufacturer’s recommendations (Abcam, UK).

### Western blotting

As previously described [38], proteins were extracted with protein lysate, and a BCA protein assay reagent kit (Beyotime Biotechnology, Shanghai, China) was used to detect their concentrations. Proteins were subjected to electrophoresis on sodium dodecyl sulfate (SDS)– polyacrylamide gels and then the target proteins were transferred onto nitrocellulose membranes. Subsequently, the membranes were blocked for 2 h at room temperature with 5% (wt/vol) milk in TBS with 0.05% (wt/vol) Tween-20. Next, The membranes were washed in tris buffered saline with Tween 20 (TBST) for three times and incubated overnight at 4°Cwith specific primary antibodies. Antibodies for p-p38 (4511S), p38 (9212S), p-JNK (4668S), JNK (9252S), p-ERK (9101S), ERK (9102S), and β-Tubulin (2146S) were obtained from Cell Signaling Technology (Danvers, MA, USA). Then the membranes were washed with TBST three times and were incubated for 2 h at room temperature with horseradish peroxidase-conjugated secondary antibody. After being washed in TBST, protein bands were detected with an enhanced chemiluminescence (ECL) detection kit (GE Health, USA), and quantified by densitometry (Tanon-4500).

### Statistical analysis

All data were presented as the mean ± standard deviation (SD). Statistical analyses were performed with SPSS 18.0 software. One-way analysis of variance was used to analyze the differences among multiple groups. *P* value < 0.05 was considered to be statistically significant.

## Results

### Body weights and organ coefficients

**Fig. 1:**
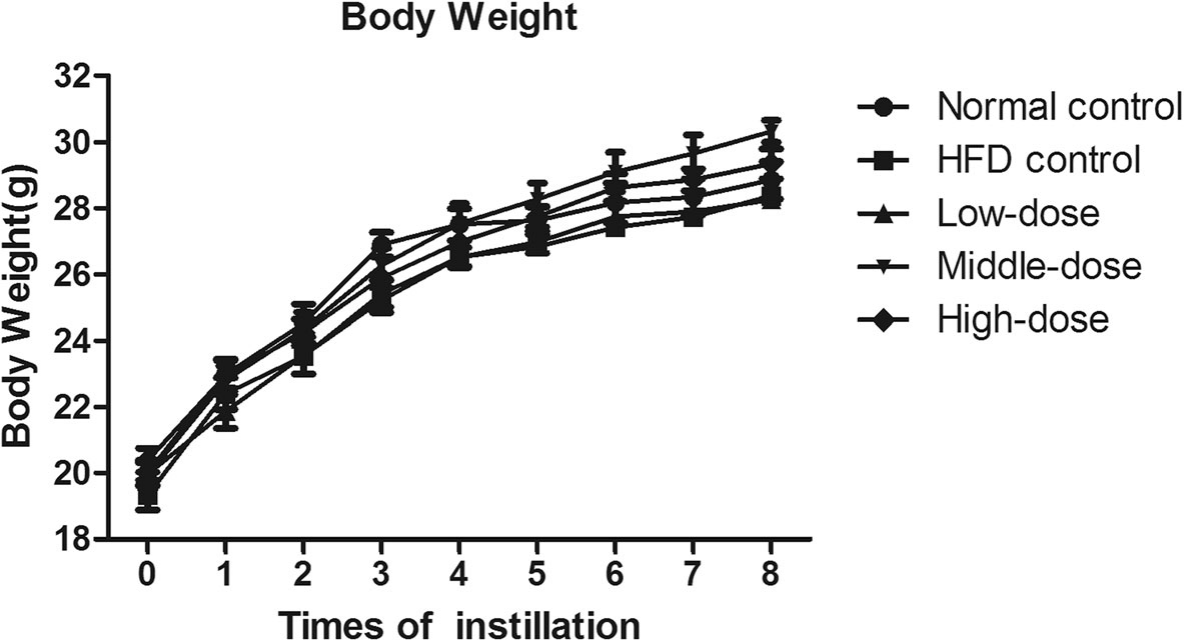
Effect of coal-fired PM_2.5_ on body weight (*n* = 8)

**Fig. 2:**
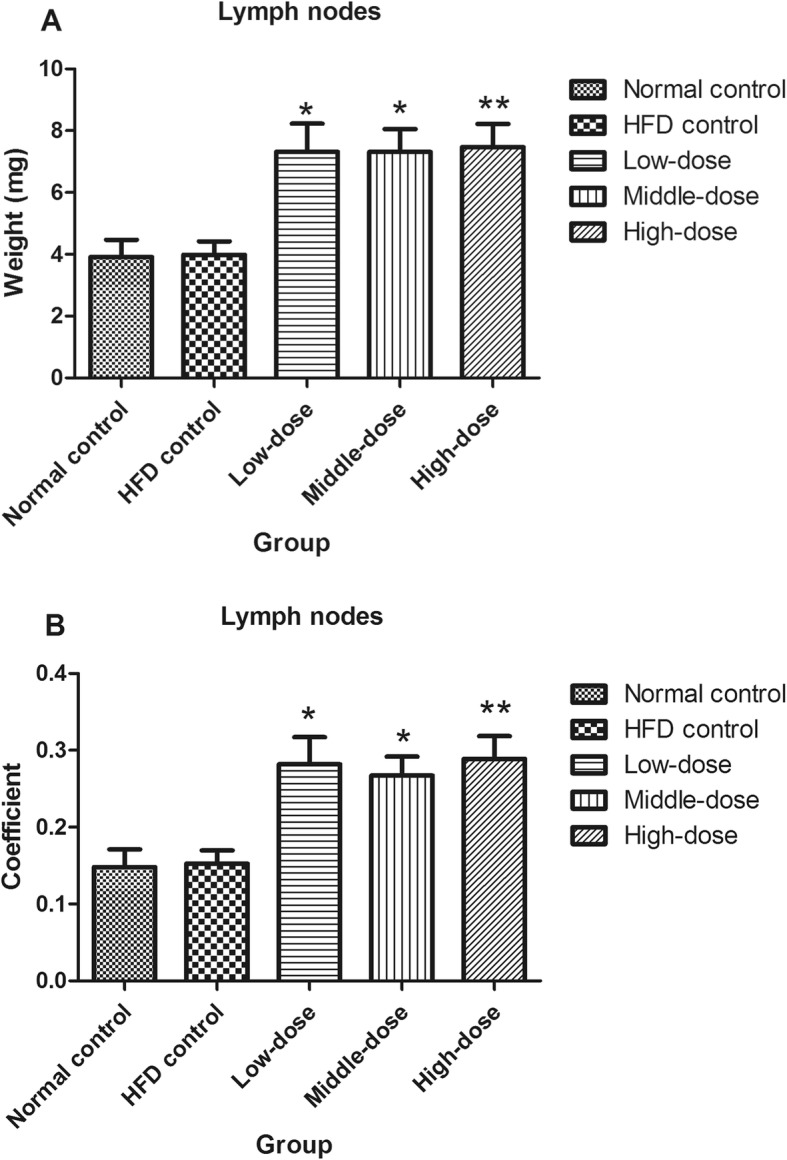
Effects of coal-fired PM_2.5_ on mediastinal lymph nodes in terms of (**a**) weight, and (**b**) organ coefficient (compared with HFD control mice, *P < 0.05, **P < 0.01, *n* = 8)

After 8 weeks of PM_2.5_ exposure, there was no significant difference in body weights among the experimental groups (Fig. 1). There were also no significant differences in the mediastinal lymph-node weights or coefficients between the normal control group and the HFD control group. However, after 8 weeks of treatment with PM_2.5_, HFD-fed ApoE^−/−^ mice had significantly increased mediastinal lymph-node weights and coefficients compared with those of HFD control mice (Fig. 2). Compared with the measured parameters in HFD control mice, PM_2.5_-treatment did not induce any changes in thymus, spleen, liver, or kidney weights—or in their corresponding organ coefficients—in HFD-fed ApoE^−/−^ mice (not shown).

### Histopathology

**Fig. 3:**
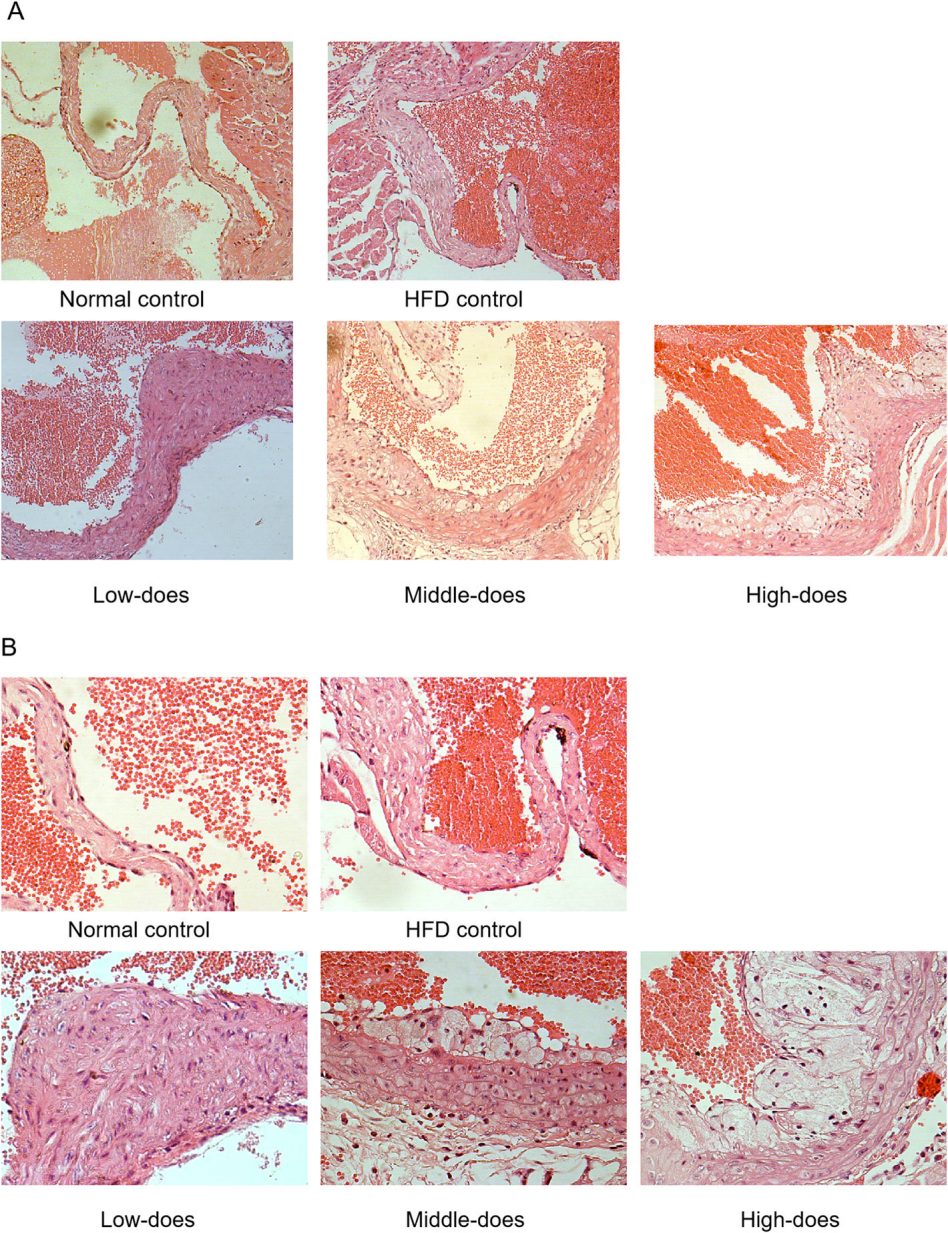
Histological assessment of ApoE^−/−^ mice aortas (**a**) 200x magnification and (**b**) 400x magnification (H&E staining)

To examine whether coal-fired PM_2.5_ promotes the formation of atherosclerosis in ApoE^−/−^ mice, we exposed them to coal-fired PM_2.5_ or PBS for 8 weeks. Cross-sections of aortas were stained with H&E (Fig. 3). In the normal control group, the intimal structure was well-organized and intact. However, the intima was markedly thickened in the HFD control group, and some foam cells were located in the subendothelial layer. Moreover, PM_2.5_ treatment exacerbated HFD-induced atherosclerosis. Cross-sections of the PM_2.5_-treated mouse aortas showed atherosclerotic lesions, intimal thickening, fibrous cap formation, and accumulation of foam cells, indicating that coal-fired PM_2.5_ promoted the formation of atherosclerosis in mice.

### The levels of atherosclerosis-related proteins

**Fig. 4:**
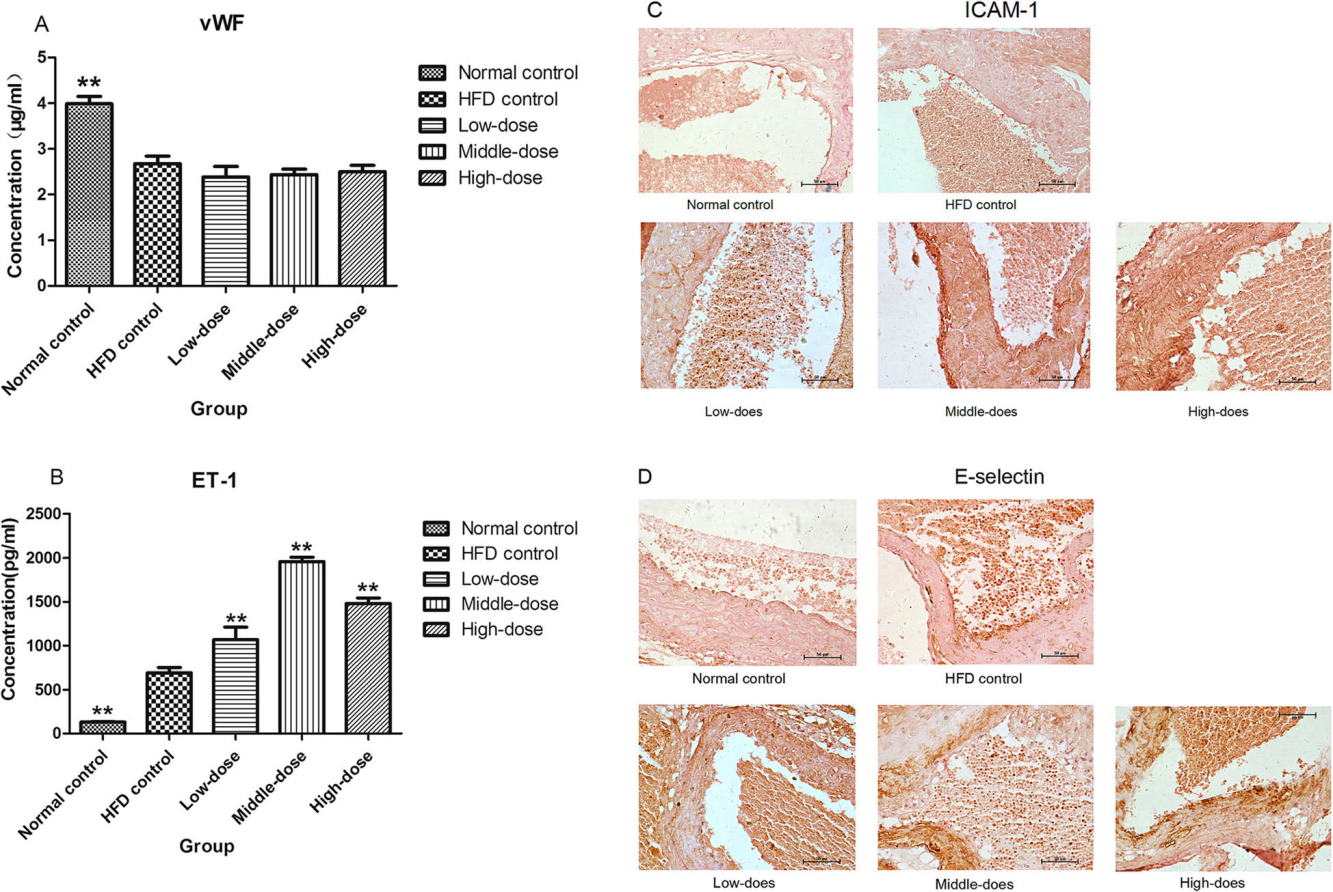
Effect of coal-fired PM_2.5_ on the levels of proteins (**a**) vWF, (**b**) ET-1, (**c**) ICAM-1 (the brown areas are ICAM-1-positive cells, 400x magnification), and (**d**) E-selectin (the brown areas are E-selectin-positive cells, 400x magnification); compared with HFD control mice, *P < 0.05, **P < 0.01, *n* = 8

Compared with the HFD control group, the vWF levels in blood plasma of PM_2.5_-treatment groups were not increased, but were significantly increased in the normal control group (Fig. 4a). This finding indicated that HFD inhibited the level of vWF in ApoE^−/−^ mice.

The levels of ET-1 in the plasma were significantly increased in PM_2.5_-treatment groups compared with the HFD control group, whereas they were lower in normal control group compared to the HFD control group (Fig. 4b). This finding indicated that PM_2.5_ increased the expression of ET-1 in ApoE^−/−^ mice.

Immunohistochemistry revealed a slight increase in the expression of ICAM-1 in the HFD control group relative to that in the normal control group. In addition, PM_2.5_ treatment significantly increased ICAM-1 expression compared with that in the HFD group (Fig. 4c).

E-selectin expression was also examined by immunohistochemistry (Fig. 4d). There were no visible E-selectin-positive cells in aortas from normal control mice, while HFD significantly increased E-selectin expression. Treatment with PM_2.5_ plus HFD markedly increased E-selectin expression in mouse aortas.

### Phosphorylation levels of components of MAPK signaling pathways

**Fig. 5:**
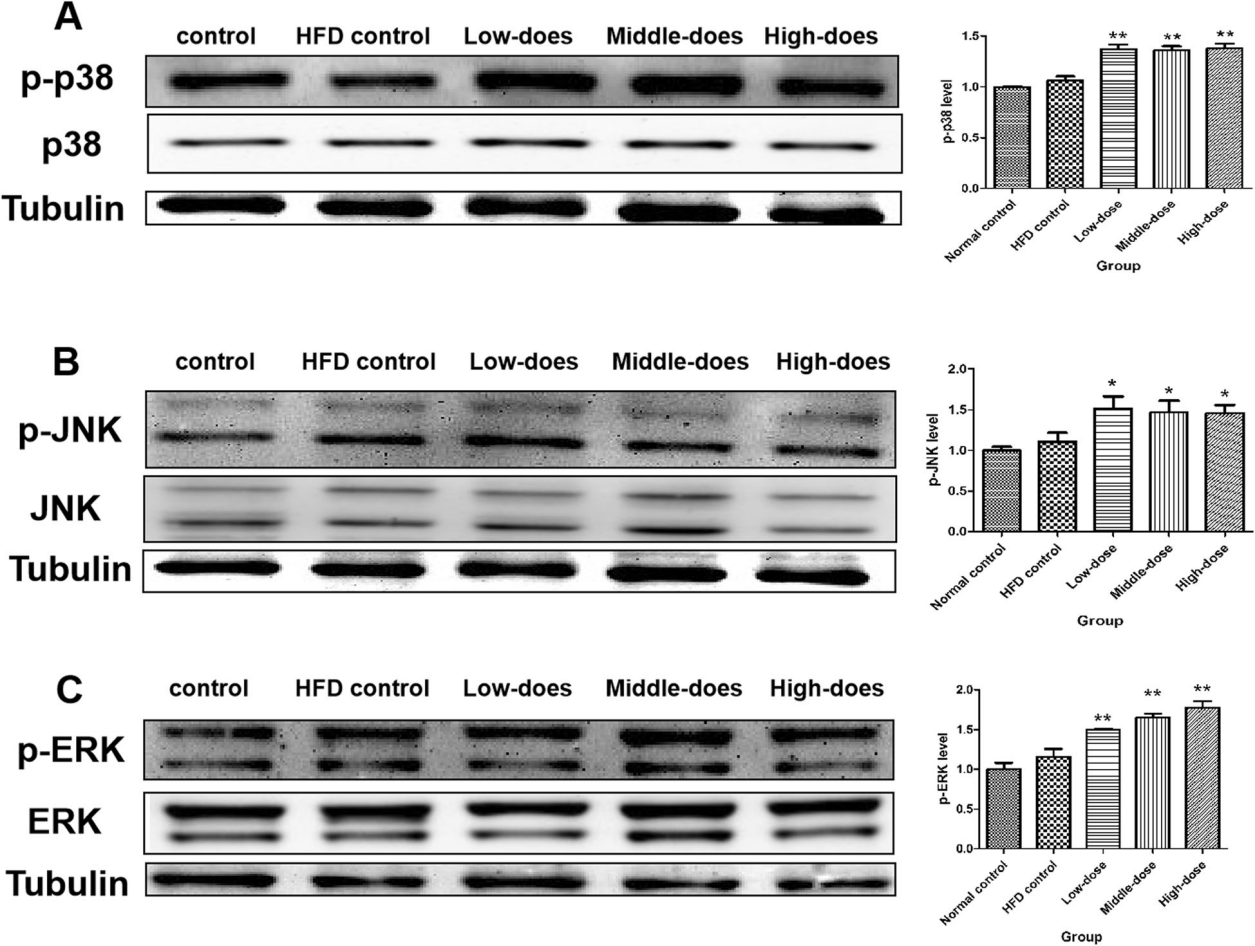
Effects of coal-fired PM_2.5_ on MAPK signaling pathways (**a**) p-p38, (**b**) p-JNK, and (**c**) p-ERK (tubulin was considered as an internal control; compared with HFD control mice, *P < 0.05, **P < 0.01, *n* = 3)

To further investigate the mechanism of PM_2.5_-induced atherosclerosis, activation of MAPK signaling pathways was examined. As shown in Fig. 5, the phosphorylation levels of p38 MAPK, ERK1/2, and JNK in PM_2.5_-treatment ApoE^−/−^ mice were significantly increased compared with those in HFD control mice. This finding indicated that coal-fired PM_2.5_ increased the phosphorylation levels of p38, ERK1/2, and JNK in mouse aortas.

## Discussion

PM_2.5_ presumably exerts atherogenic effects by inducing endothelial damage, mitochondrial injury, inflammatory responses, and oxidative stress [21, 23, 28, 39, 40]. In our present study, PM_2.5_-treatment significantly increased lymph-node weights and coefficients compared with those of HFD control group. It indicated that in the process of inflammation induced by coal-fired PM_2.5_ immunocytes might be involved in and play role in injury or recovery. So further studies are needed to elucidate potential mechanisms underlying this phenomenon.

Epidemiological and experimental studies have suggested that PM_2.5_ exposure is a risk factor which promotes the development of AS [41], and lipids accumulation and fibrous plaques formation in arteries are characters of AS [42, 43]. In our present study, cross-sections of PM_2.5_-treated mouse aortas exhibited atherosclerotic lesions, intima thickening, fibrous cap formation, and foam cell accumulation, indicating that coal-fired PM_2.5_ promoted the formation of atherosclerosis in mice.

The expression of adhesion molecules on the endothelium increased during the progression of AS, which involve the recruitment of monocytes into the circulation and trans-endothelial migration [44]. Moreover, eficiencies of adhesion molecules would inhibit monocyte migration and AS formation in mice [36].

vWF is a large glycoprotein [45] that can be produced in endothelial cells and megakaryocytes [46–48]. vWF involves the adhesion of platelets to endothelial cells [47], which is associated with the formation of thrombus and atherosclerosis [49, 50]. But It is still controversial whether vWF plays a key regulatory role in the AS formation induced by coal-fired PM_2.5_. A study showed PM_2.5_ could increase vWF in old people, but another stuy showed PM_2.5_ could decrease vWF level in rats [51]. In our study, there was no significant difference in the vWF levels between the PM_2.5_-treatment mice and the HFD control mice. Our result was consisted with those in the previous study [52], which showed PM_2.5_ could not increase vWF in young adults. Different results may caused by different species and different physical conditions. Therefore, further studies are needed to elucidate whether vWF is really involved in the process of AS formation induced by PM_2.5_ or not.

ET-1 is a vasoconstrictor peptide that is synthesized by endothelial cells of the vascular wall [53] and by macrophages [54], and has been demonstrated to be a potent vasoconstrictor [55–58]. ET-1 directly affects blood vessels and the heart [59] and is implicated in many forms of cardiovascular disease [60–63]. Studies have demonstrated that the overexpression of ET-1 exacerbates HFD-induced AS in ApoE^−/−^ mice [64, 65]. However, it remains unclear how increased ET-1 expression exacerbates atherosclerotic progression in HFD-fed ApoE^−/−^ mice [64]. In the present study, we found that coal-fired PM_2.5_ elevated the expression of ET-1 in mice plasma. We can not obtain a very ideal dose-response relationship for this index, there are two reasons may induce such condition. The first is that in the experiment only several mice were used for each group and there must be some sampling error which may influence the representativeness of mean for the population. Second, there is no liner relationship between the dose and the response for this index.

ICAM-1 is a transmembrane glycoprotein [66] and is typically expressed on the surface of endothelial and immune cells [67]. Cell-adhesion molecules, such as ICAM-1, involve in binding and recruitment of circulating leukocytes to the vascular endothelial cells and further migration into subendothelial spaces, which are primary processes of AS [68, 69]. Hence, ICAM-1 may play a key role at the initial stage of AS [70–73]. In the present study, we found that coal-fired PM_2.5_ increased the expression of ICAM-1 in ApoE^−/−^ mouse aortas. In addition, a soluble form of ICAM-1 has been found in plasma, which may be involved in the progression of AS [74].

E-selectin is a transmembrane glycoprotein [75] and expressed exclusively on the surface of endothelial cells [76, 77]. E-selectin is important for the initial rolling interaction [78–80] and subsequent adhesion [81] of leukocytes in the inflamed endothelium, as well as for the transmigration of inflammatory cells to inflammation sites [76], which are critical events in the initiation of AS [82]. Moreover, monocytes are recruited to lipid-rich plaques mediated by E-selectin during the progression of AS [83]. E-selectin is a hallmark of atherogenesis [84–86] and is implicated in the destabilization of atherosclerotic plaques [87]. It has been reported that E-selectin is mostly absent in the healthy endothelial cells but is apparently upregulated in aberrant endothelia that are covered with atherosclerotic plaques in mice and humans [86]. E-selectin is associated with PM_2.5_ measurements at the day of blood drawing [88]. Our present study found that coal-fired PM_2.5_ promoted E-selectin expression, indicating that PM_2.5_ may aggravate arteriosclerosis by inducing upregulation of E-selectin. Taken together, the PM_2.5_-induced the changes of multiple proteins expression in the present study suggest that these proteins may link coal-fired PM_2.5_ exposure with the formation of atherosclerosis.

MAPK signaling pathways are a series of parallel cascades of serine/threonine kinases [31] that transduce extracellular signals into cells and induce cellular biological responses [89]. MAPK signaling pathways play an important role in regulating the cardiovascular system [90], and they also influence the formation and development of atherosclerosis [91, 92]. PM_2.5_ increases ET-1 levels and markedly upregulates p-p38 MAPK expression in vascular smooth muscle cells [93]. Studies have shown that cigarette-smoke extracts upregulate the ICAM-1 and E-selectin expressions via phosphorylation of JNK and ERK pathways [94, 95]. Moreover, a study demonstrated that PM_2.5_ increases the expression of ICAM-1 in human endothelial cells via ERK pathway [96]. In the present study, western blotting showed that coal-fired PM_2.5_ induced phosphorylation of p38, JNK, and ERK kinases in mouse aortas. Hence, MAPK signaling pathways may partially link coal-fired PM_2.5_ exposure with upregulation of ET-1, ICAM-1 and E-selectin.

## Conclusion

Coal-derived PM_2.5_ exacerbated the formation of atherosclerosis in mice, increased the expression levels of atherosclerosis-related proteins (ET-1, ICAM-1 and E-selectinin) in mice serum and promoted the phosphorylation of proteins relevant to MAPK signaling pathway. Therefore, We postulate that MAPK signaling pathway may play a role in the atherosclerosis pathogenesis induced by coal-derived PM_2.5_. More researches need to be conducted on the relationship between atherosclerosis-related proteins and MAPK signaling pathway and the underlying mechanism needs to be elucidated further in the future.

## Supplementary information

**Additional file 1.** Highlights

## Data Availability

All data generated or analysed during this study are included in this published article.

## Abbreviations

AS: Atherosclerosis
ApoE-/-: Apolipoprotein-E knockout
BSA: Bovine serum albumin
CVD: Cardiovascular disease
DAB: Diaminobenzidine
ECL: Enhanced chemiluminescence
ELISA: Enzyme-linked immunosorbent assay
ERK: Extracellular signal-regulated kinase
ET-1: Endothelin-1
FBS: Fetal bovine serum
H&E: Hematoxylin and eosin
HFD: High-fat diet
HRP: Horseradish peroxidase
ICAM-1: Intercellular adhesion molecule-1
JNK: c-Jun N-terminal kinase
MAPK: Mitogen-activated protein kinase
PBS: Phosphate buffer saline
PM2.5: Fine particulate matter
SD: Standard deviation
SDS: Sodium dodecyl sulfate
SLE: Systemic lupus erythematosus
TBS: Tris buffered saline
TBST: Tris buffered saline with Tween 20
vWF: von Willebrand factor
WHO: World Health Organization
