# Supplementary material for: Effects of coal-fired PM2.5 on the expression levels of atherosclerosis-related proteins and the phosphorylation level of MAPK in ApoE−/− mice
Source: BMC Pharmacol Toxicol. 2020 May 8;21:34. doi: 10.1186/s40360-020-00411-8 (PMC7206822; doi:10.1186/s40360-020-00411-8)
Supplement: Supplementary file 1 — Additional file 1. Highlights [file 40360_2020_411_MOESM1_ESM.doc]

Highlights

Coal-fired PM_2.5_ exacerbated atherosclerosis induced by a high-fat diet in apolipoprotein-E knockout (ApoE^-/-^) mice.

Coal-fired PM_2.5_ increased the protein levels of Endothelin-1 (ET-1), intercellular adhesion molecule-1 (ICAM-1), and E-selectinin in aortic tissues of ApoE^-/-^ mice.

Coal-fired PM_2.5_ promoted the phosphorylation of p38, c-Jun N-terminal kinase (JNK), extracellular signal-regulated kinase (ERK) in aortic tissues of ApoE^-/-^ mice.
